# The impact of interactive motor-cognitive dual tasking on brain activation, functional connectivity, and behavioral performance in healthy adults: an fNIRS study

**DOI:** 10.3389/fnhum.2025.1464617

**Published:** 2025-06-25

**Authors:** Xiaohan Li, Lifeng Tang, Yuting Zhang, Lin Ye, Lifeng Zhou, Min Tang

**Affiliations:** ^1^Department of Neurological Rehabilitation, Ningbo Rehabilitation Hospital, Ningbo, China; ^2^Faculty of Rehabilitation, Gannan Medical University, Ganzhou, China; ^3^Faculty of Marine, Ningbo University, Ningbo, China; ^4^Faculty of Health Service and Health, Ningbo College of Health Sciences, Ningbo, China

**Keywords:** fNIRS, interactive dual task, brain activation, functional connectivity, lateralization

## Abstract

**Objective:**

This study aimed to explore how varying levels of interactive motor-cognitive dual task difficulty affect brain activation, functional connectivity (FC), and behavioral performance in healthy adults using functional near-infrared spectroscopy (fNIRS).

**Methods:**

We recruited 28 healthy participants to perform interactive motor-cognitive dual task at three difficulty levels: easy task (ET), medium task (MT), and difficult task (DT). The tasks involved walking while simultaneously engaging in cognitive challenges. A continuous-wave fNIRS system was used to collect fNIRS data during the task, focusing on 10 regions of interest (ROIs): left/right prefrontal cortex (LPFC/RPFC), left/right dorsolateral prefrontal cortex (DLPFC/DRPFC), left/right premotor cortex (LPMC/RPMC), left/right sensorimotor cortex (LSC/RSC), and left/right motor cortex (LMC/RMC). Simultaneously, the subjects’ gait data during walking were collected using an Inertial Measurement Unit (IMU) sensor, and their cognitive performance was recorded by the researchers.

**Results:**

Statistical analysis revealed statistically significant differences in the mean HbO levels among the three groups for the DRPFC, LPMC/RPMC, RSC, and LMC/RMC regions. Additionally, significant differences were found in the activation of channels 3, 18, 24, 25, 28, and 29 across the three groups. The group-averaged FC in the DT (0.61 ± 0.21) was significantly higher than that in the ET (0.46 ± 0.21, *P* = 0.023). ROI-to-ROI FC analysis showed significant differences among the three groups in the LSC∼RPMC, RPMC∼RSC, and RSC∼RMC connections. The lateralization index (LI) ranged from 0.10 to 0.35, indicating a predominant right-brain lateralization during the interactive motor-cognitive dual task. Additionally, compared to the MT, both speed and stride length, as well as cognitive performance, were lower during the DT.

**Conclusion:**

We found that increased task difficulty heightened activation in the premotor and motor cortices, with a tendency toward right hemisphere dominance. Higher task difficulty also strengthened FC, particularly in motor-related regions, indicating greater neural coordination. Behaviorally, participants exhibited slower gait parameters and reduced cognitive performance as task complexity increased, highlighting the impact of dual-task interference.

## 1 Introduction

Most daily activities involve managing motor–cognitive tasks while processing external information, such as crossing a street while observing traffic or carrying a cup of tea while thinking about a shopping list. These motor–cognitive interactions are referred to as motor–cognitive dual task performance (Wollesen and Voelcker-Rehage, 2014). Motor and cognitive dual task training can be categorized into sequential and simultaneous approaches. Sequential motor-cognitive training involves motor and cognitive training occurring at separate times, either before or after physical exercises or on different days (Herold et al., 2018; Tait et al., 2017). Simultaneous motor-cognitive training, on the other hand, involves training both motor and cognitive tasks simultaneously (Lauenroth et al., 2016). A recent review found that simultaneous training significantly improves cognitive performance across various populations, whereas evidence regarding the effectiveness of sequential training remains inconclusive (Tait et al., 2017). Furthermore, the optimal interval between motor and cognitive exercises in sequential training is yet to be determined (Herold et al., 2018; Tait et al., 2017). These findings suggest that simultaneous motor-cognitive training is a more promising and time-efficient approach to enhancing cognitive functions compared to sequential training regimens.

Interactive motor-cognitive dual task is a type of simultaneous motor-cognitive training, in which cognitive task is “incorporated” into the motor task. The cognitive task is a relevant prerequisite to successfully solve the motor-cognitive task (e.g., walking to certain cones in a predefined order or dancing) (Schott, 2015). Several studies have shown that interactive motor-cognitive dual task has many advantages (Herold et al., 2018; Laguë-Beauvais et al., 2015; Plummer et al., 2015; Shams and Seitz, 2008; Yogev-Seligmann et al., 2012). Firstly, interactive motor-cognitive dual task is closer to daily life situations (Herold et al., 2018). For example, it is unlikely that an older person habitually solves an arithmetic task during walking, but it is likely that he/she walks through the supermarket while remembering what goods to buy and where to find those. Secondly, if the cognitive task is incorporated into the motor task, no prioritization effects would occur (Plummer et al., 2015). Such prioritization effects (giving priority either to the cognitive or the motor task) are known to influence motor and cognitive performance (Laguë-Beauvais et al., 2015; Plummer et al., 2015; Yogev-Seligmann et al., 2012). A further advantage of interactive motor-cognitive dual task could be that multiple sensory systems are stimulated due to the execution and control of the cognitive task and the motor task at the same point of time (Shams and Seitz, 2008).

Functional near-infrared spectroscopy (fNIRS), a non-invasive neuroimaging technique, has emerged as a promising tool for investigating brain activation and functional connectivity (FC) (Leff et al., 2011). By measuring fluctuations in oxygenated hemoglobin (HbO) and deoxygenated hemoglobin (HbR) concentrations in response to neuronal activity, fNIRS offers unique insights into the functional dynamics of the brain. FC refers to the interactions between different regions of the cerebral cortex during neurophysiological processes, highlighting important connections among distinct cortical areas (Chen et al., 2022; Liu et al., 2021). Previous fNIRS studies have successfully applied dual task paradigms in both healthy individuals (Yao et al., 2024) and patients, including hypertension (Yao et al., 2024), cognitive impairment (Cao et al., 2022), stroke (Hermand et al., 2019), Parkinson’s disease (Ranchet et al., 2020), and schizophrenic (Ji et al., 2020; Xia et al., 2022; Yang et al., 2020).

Despite the potential of interactive motor-cognitive dual tasking, gaps remain in the current literature. Many of these studies have primarily focused on activation in the prefrontal cortex (PFC), a region known for its involvement in executive functions and cognitive control (Lapanan et al., 2023; Miura et al., 2024). However, research targeting other brain regions associated with motor and cognitive processes, such as the motor cortex, remains scarce. Furthermore, the impact of varying task difficulty on brain activation and FC across these regions is not yet fully understood. Although some studies have reported that increased task difficulty leads to heightened PFC activity (Chen et al., 2022), it is still unclear whether similar patterns are observed in other cortical areas, especially during tasks requiring both motor and cognitive engagement. Thus, further investigation is necessary to elucidate how task complexity influences brain activation beyond the PFC and whether this extends to changes in FC across multiple brain regions.

Given the advantages of interactive motor-cognitive dual task, this study aimed to investigate the neural mechanisms underlying this approach. Specifically, we used fNIRS to assess how varying levels of task difficulty affect brain activation, FC, and both motor (walking) and cognitive performance in healthy adults. By analyzing changes in brain activity and connectivity across different task conditions, we aimed to understand how the brain adapts to increased cognitive and motor demands. Our hypothesis posits that (1) as task difficulty increases, there will be a corresponding increase in brain activation across multiple cortical regions, accompanied by heightened FC between task-relevant regions; (2) behavioral performance will show differential adaptation based on task difficulty.

## 2 Materials and methods

### 2.1 Participants

We recruited 28 healthy subjects (age: 22.53 ± 3.46 years, height: 165.71 ± 6.31 cm, weight: 61.97 ± 9.48 kg, education: 14 ± 2.35 years) from Ningbo Rehabilitation Hospital participated in the study, including 14 men and 14 women. Inclusion criteria: participants must have no cognitive impairment, as indicated by a Mini-Mental State Examination (MMSE) score of ≥ 24 (Tombaugh and McIntyre, 1992); voluntary consent to participate in the experiment; absence of cardiovascular, respiratory, musculoskeletal, or neurological diseases; the ability to walk independently; and right-handedness. Right-handed participants were specifically chosen to minimize variability in brain activation patterns, as handedness has been shown to influence functional lateralization and cortical activation in motor and cognitive tasks (Shirzadi et al., 2020). Additionally, while some of the participants were below the legal age of adulthood in certain jurisdictions, they were all university students or young adults with fully developed cognitive and motor capabilities. This age group was selected to ensure homogeneity in cognitive function and neuroplasticity, reducing potential confounding effects that might arise from age-related cognitive decline or neurodevelopmental differences. Exclusion criteria: visual impairment and use of medications affecting the nervous system within the last six months.

The required sample size was calculated using G*power software (version 3.1, University of Kiel, Kiel, Germany) (Kang, 2021). The statistical test used in this study was a one-way repeated measures ANOVA. The input parameters were set as follows: effect size = 0.4, α error probability = 0.05, power (1—ß error probability) = 0.85, and number of groups = 3. Based on these calculations and considering an anticipated rate of approximately 15%, 28 participants were recruited for this study.

Before the fNIRS experiment, the purpose, procedures, fNIRS system, and potential risks of the study were informed in detail with the subjects and their family members to ensure participants could make an informed decision regarding their involvement in the experiment. All participants provided written informed consent before data collection. The experimental protocol was approved by the Institutional Review Board of Ningbo Rehabilitation Hospital (2022-03-G2). All experimental procedures were performed in accordance with the latest guidelines and regulations of the Declaration of Helsinki.

### 2.2 Experimental protocol

In this study, participants performed an interactive motor-cognitive dual task, which involved walking while completing a variant of the color-word Stroop task. Stroop task is one of the most widely used tasks in cognitive function (Qi et al., 2024). In most traditional versions of the Stroop task, words are displayed on a screen, and participants are instructed to quickly and accurately identify the font color of the words (Qi et al., 2024; Riedel et al., 2024; Wollesen et al., 2016). To increase ecological validity and better approximate real-life situations that require simultaneous motor and cognitive engagement, we adapted the Stroop task into a dynamic, walking-based format.

**FIGURE 1:**
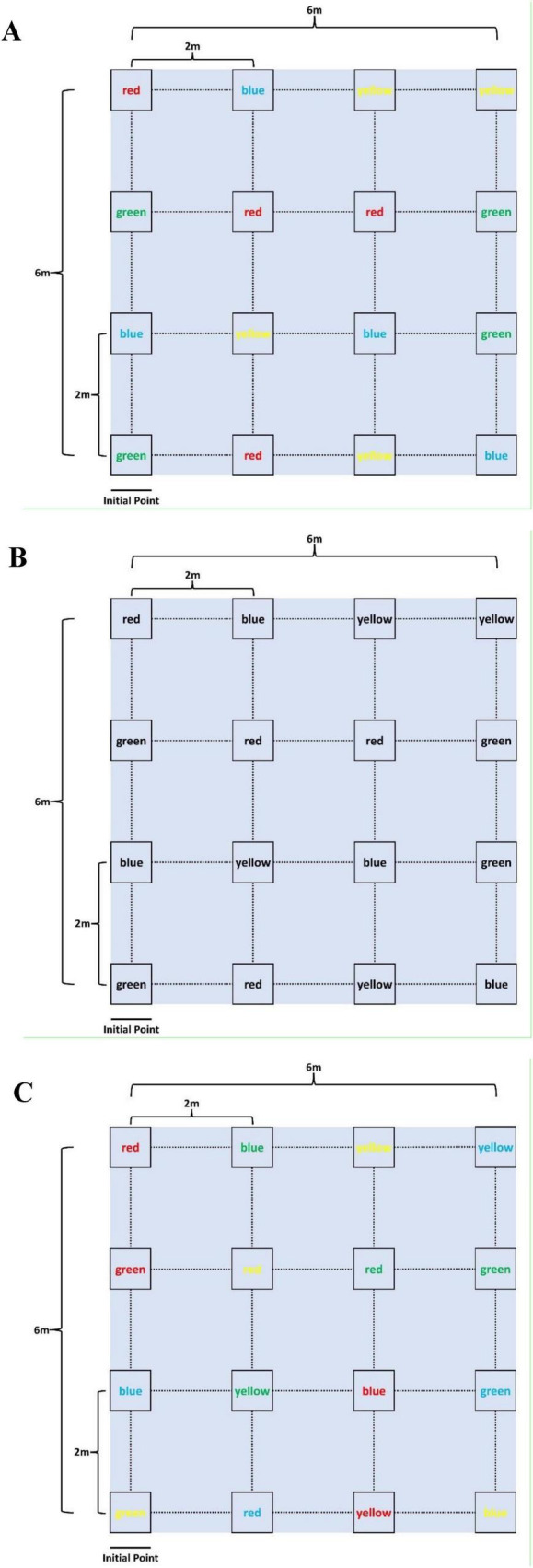
Interactive motor-cognitive dual task diagram. **(A)** Easy task (ET), **(B)** medium task (MT), **(C)** difficult task (DT).

A 4 × 4 matrix of color words (red, blue, green, and yellow) was placed on the ground in a 6 × 6 meter grid, with each row and column spaced 2 meters apart (Figure 1 and Supplementary Video). Each word was printed in a specific ink color, which could either match or differ from the written word (e.g., the word “red” might be printed in green ink). This created a Stroop-like cognitive interference task, requiring participants to focus on both the written word and its ink color. Participants were instructed to walk at a comfortable pace in an initial point through the grid, guided by the researcher’s verbal instructions. For example, if the researcher instructed participants to find the word “red” displayed in green font, they needed to locate the corresponding card within the matrix (i.e., the card showing the word “red”). After identifying the target, the participant would move to its location and await the next instruction. Subsequent instructions required participants to continue from their current position, searching for the next target without returning to the initial point. During the ET and MT tasks, participants were provided with four possible word options at each instruction, and they were required to select a different word each time, ensuring no repetitions. To reduce the potential influence of learning and adaptation, the three tasks were presented in a randomized order. Each trial lasted one minute, with the card layout reshuffled for every test to further minimize any learning effects. To ensure participants remained engaged and to maintain measurement accuracy, a one-minute rest period was provided between tasks to prevent fatigue.

Three levels of difficulty were introduced to the interactive motor-cognitive dual task:

1. Easy task (ET): The font color and the word itself were congruent, presenting no conflict (e.g., the word “red” was printed in red ink, as shown in Figure 1A). This task required minimal cognitive effort, focusing mainly on motor activity.
2. Medium task (MT): The words were printed in black ink, removing the color-coding element. Participants had to rely solely on reading and interpreting the text for the cognitive challenge (Figure 1B), increasing the cognitive demand.
3. Difficult task (DT): The font color and the word were incongruent (e.g., the word “red” was printed in green ink, as shown in Figure 1C), creating a Stroop-like interference. This task required participants to suppress the automatic tendency to respond to the font color, significantly increasing the cognitive load.

### 2.3 Data acquisition

#### 2.3.1 fNIRS data acquisition

**FIGURE 2:**
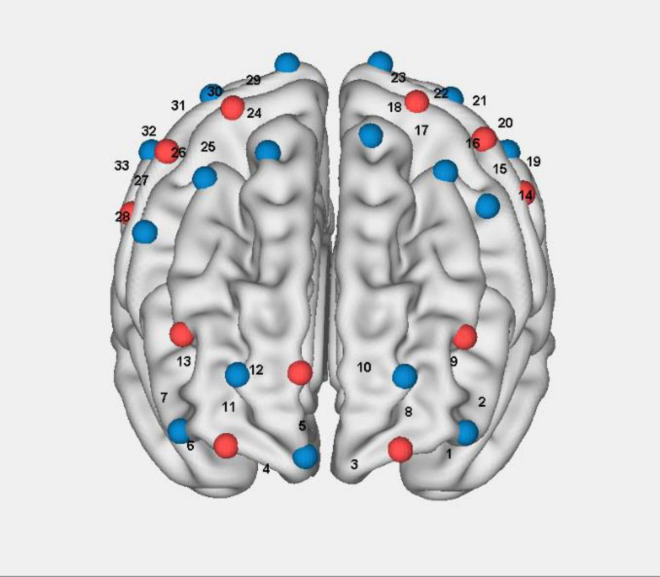
Schematic of fNIRS channel arrangem.

A continuous-wave fNIRS system (Nirsmart, Danyang Huichuang Medical Equipment Co., Ltd., China) was employed in this study to measure cortical hemodynamic responses associated with interactive motor-cognitive dual tasks. This system has been widely utilized in cognitive and motor neuroscience research, demonstrating its reliability in detecting functional brain activity during movement-based tasks (Chen et al., 2022; Li et al., 2020; Li et al., 2023; Miura et al., 2024; Ou et al., 2024). The system operates at two wavelengths (760 and 850 nm) and collects data at a sampling rate of 11 Hz. The setup consisted of 33 channels, formed by 17 source optodes and 11 detector optodes, which were symmetrically placed over both hemispheres following the 10/20 international electrode placement system (Figure 2). These channels covered multiple brain regions of interest (ROIs), including the left and right prefrontal cortex (LPFC/RPFC), dorsolateral prefrontal cortex (DLPFC/DRPFC), premotor cortex (LPMC/RPMC), sensorimotor cortex (LSC/RSC), and motor cortex (LMC/RMC) (Table 1). The selection of these ROIs was based on Brodmann areas (BA) and anatomical references relevant to motor and cognitive processes. To ensure standardized spatial localization, the acquired optode coordinates were transformed into Montreal Neurological Institute (MNI) coordinates and mapped onto the MNI standard brain template using the NirSpace software (Danyang Huichuang Medical Equipment Co., Ltd., China) (Li et al., 2023). The emitter-detector distance was 3 cm. Prior to each recording, a NIR gain quality check was performed to ensure moderate data acquisition, avoiding both under-gained and over-gained conditions.

**TABLE 1:** The MNI coordinates and ROI corresponding to the measurement channels.

Channel number	S-D	x	Y	z	ROI
CH 1	S1-D1	−36.909	61.405	−15.8	LPFC
CH 2	S1-D3	−49.314	52.632	−0.63518	DLPFC
CH 3	S2-D1	−10.808	69.15	−16.099	LPFC
CH 4	S2-D2	15.535	68.772	−15.143	RPFC
CH 5	S2-D4	2.7718	70.555	−1.8321	RPFC
CH 6	S3-D2	40.094	60.664	−14.239	RPFC
CH 7	S3-D5	49.805	51.245	−0.16077	DRPFC
CH 8	S4-D1	−27.606	68.604	−1.8545	LPFC
CH 9	S4-D3	−38.615	59.586	16.23	DLPFC
CH 10	S4-D4	−13.138	71.129	16.147	DLPFC
CH 11	S5-D2	30.461	66.68	−2.1316	RPFC
CH 12	S5-D4	16.841	69.921	16.074	DRPFC
CH 13	S5-D5	41.077	58.47	14.91	DRPFC
CH 14	S6-D6	−59.254	−6.0835	47.576	LSC
CH 15	S6-D7	−50.97	−3.9333	56.165	LPMC
CH 16	S7-D7	−41.861	−3.5054	63.365	LPMC
CH 17	S7-D8	−31.739	−4.1798	68.142	LPMC
CH 18	S8-D8	−19.98	−4.271	77.095	LPMC
CH 19	S9-D6	−59.312	−27.448	53.239	LSC
CH 20	S9-D7	−52.518	−26.987	63.059	LSC
CH 21	S10-D7	−43.073	−26.507	68.706	LSC
CH 22	S10-D8	−32.398	−25.435	73.347	LMC
CH 23	S11-D8	−20.701	−26.071	77.376	LMC
CH 24	S12-D9	22.861	−0.59311	74.834	RPMC
CH 25	S13-D9	35.358	−1.364	65.886	RPMC
CH 26	S13-D10	44.834	−1.0439	60.313	RPMC
CH 27	S14-D10	56.272	−1.8706	51.544	RPMC
CH 28	S14-D11	62.484	−2.709	39.983	RSC
CH 29	S15-D9	22.551	−24.71	76.87	RMC
CH 30	S16-D9	34.414	−23.983	72.973	RMC
CH 31	S16-D10	44.927	−23.861	67.31	RSC
CH 32	S17-D10	55.443	−24.5	58.323	RSC
CH 33	S17-D11	63.917	−27.722	48.956	RSC

ROI, regions of interest; LPFC/RPFC, left/right prefrontal cortex; DLPFC/DRPFC, left/right dorsolateral prefrontal cortex; LPMC/RPMC, left/right premotor cortex; LSC/RSC, left/right sensorimotor cortex; LMC/RMC, left/right motor cortex.

#### 2.3.2 Behavioral data acquisition

The Inertial Measurement Unit (IMU) sensor was positioned approximately 5 cm above the subject’s medial ankle to collect walking performance data during the task. The walking performance index included step speed, step length, and stride width. Researchers recorded the number of words each subject found during each task as an indicator of cognitive performance.

### 2.4 Data processing and analysis

#### 2.4.1 Preprocessing

We preprocessed the data collected using fNIRS in the preprocessing module of the NirSpark (Danyang Huichuang Medical Equipment Co., Ltd., China) software, which has been used in previous experiments (Li et al., 2020; Li et al., 2023). The preprocessing steps were as follows:

The standard deviation threshold was set to 6.0, and the amplitude threshold was set to 0.5. Motion artifacts were removed using standard deviation combined with cubic spline interpolation. Interference signals caused by heart rate, breathing rate, and Mayer waves were removed using a 0.01 to 0.2 Hz band-pass filter. Differential path-length factors were set to 6.0. Optical density was converted to blood oxygen concentration using the modified Beer-Lambert law.

#### 2.4.2 Brain activation

In this study, we selected the concentration of HbO as the target indicator of brain activation, as the HbO signals have a higher signal-to-noise ratio (Zhang et al., 2023). The steps for analyzing the HbO time-series data were:

The initial time of the hemodynamic response function (HRF) was set to −2 s and the end time to 60 s. The baseline state was retained from −2 s to 0 s, while the single block paradigm lasted from 0 s to 60 s. The generalized linear model (GLM) was used to generate an ideal HRF for each task. Experimental HRF values were compared with the ideal HRF values to determine the corresponding range. The beta value, indicating the extent of brain activation, served as an indicator for estimating the HRF prediction of the HbO signal. The mean HbO for each ROI in the task states was obtained by dividing the concentration of HbO in all channels in each ROI by the number of channels in each ROI.

#### 2.4.3 Functional connectivity

The FC matrix was computed in NirSpark using Pearson’s correlation analysis between the time series for each pair of channels. Fisher’s r-to-z transformation was conducted to improve normality. For each participant, a 33 × 33 correlation matrix was generated. The ROI-to-ROI correlation coefficients for each participant were calculated. The time series of all channel pairs were averaged for each participant, contributing to the general FC value for each participant. To ensure statistical robustness, we applied a false discovery rate (FDR) correction at *q* < 0.05 to control for multiple comparisons when analyzing connectivity data.

#### 2.4.4 Laterality index

The lateralization index (LI) evaluates interhemispheric regional activation asymmetry during interactive motor-cognitive dual task. The calculation of the LI was performed as follows:


$$\text{LI}=\frac{\text{ABS}\left(\text{HbO}\_<\mathrm{sc}>\text{R}</\text{sc}>\right)\text{ABS}\left(\text{HbO}\_\text{L}\right)}{\text{ABS}\,\left(\text{HbO}\,\_\,\text{R}\right)+\text{ABS}\,\left(\text{HbO}\,\_\,\text{L}\right)}$$

HbO_L represents the average oxygenated hemodynamic response of channels in the left hemisphere, HbO_R represents the average oxygenated hemodynamic response of channels in the right hemisphere. The value of the LI ranges between −1 and +1. An LI value of “−1” indicates complete left hemisphere dominance, while an LI value of “+1” indicates complete right hemisphere dominance (Borrell et al., 2023; Seghier, 2008).

### 2.5 Statistical analysis

Statistical analysis was performed using SPSS 27.0 software. The Shapiro–Wilk test was applied to assess data normality, and Levene’s test was used to evaluate the homogeneity of variances. Given that this study involved the same group of participants performing multiple tasks under different conditions (ET, MT, and DT), a one-way repeated measures ANOVA was chosen to analyze differences in: walking performance (speed, step length, and stride width), cognitive performance (number of words found), fNIRS data (ROI activation, channel activation, channel FC, ROI-to-ROI FC, and LI). *Post-hoc* comparisons were corrected using the Bonferroni method. FDR correction was applied to the fNIRS data. All *P*-values reported are based on two-sided tests, with values less than 0.05 considered statistically significant.

## 3 Results

### 3.1 fNIRS data

#### 3.1.1 Brain activation

**FIGURE 3:**
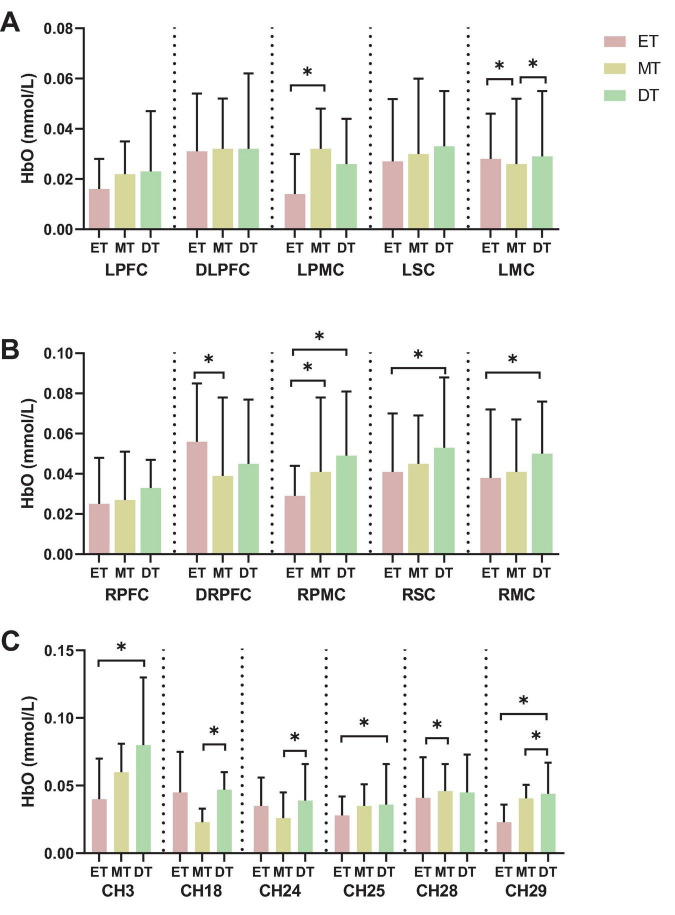
Results of brain activation. **(A)** left hemisphere activation, **(B)** right hemisphere activation, **(C)** channel activation. *P < 0.05. ET, easy task; MT, medium task; DT, difficult task; LPFC/RPFC, left/right prefrontal cortex; DLPFC/DRPFC, left/right dorsolateral prefrontal cortex; LPMC/RPMC, left/right premotor cortex; LSC/RSC, left/right sensorimotor cortex; LMC/RMC, left/right motor cortex.

The results from Figures 3A, B, which show the activation in the left and right ROIs, indicate the following: the activation of LPMC in MT (0.032 ± 0.016) was higher than in ET (0.014 ± 0.016), the activation of LMC in both DT (0.029 ± 0.026) and ET (0.028 ± 0.018) was higher than in MT (0.026 ± 0.026), the activation of DRPFC in ET (0.056 ± 0.029) was higher than in MT (0.039 ± 0.039), the activation of RPMC in both MT (0.041 ± 0.037) and DT (0.049 ± 0.032) was higher than in ET (0.029 ± 0.015), and the activation of RSC and RMC in DT (0.053 ± 0.035, 0.050 ± 0.026) were higher than in ET (0.041 ± 0.029, 0.038 ± 0.034).

Additionally, when comparing the activation of channels across the three groups, the results show significant differences in channels 3, 18, 24, 25, 28, and 29 (Figure 3C). Specifically: the activation of channel 3 and 25 in DT (0.080 ± 0.050, 0.036 ± 0.030) was higher than in ET (0.040 ± 0.030, 0.028 ± 0.014), the activation of channel 18 and 24 in DT (0.047 ± 0.013, 0.039 ± 0.027) was higher than in MT (0.023 ± 0.010, 0.026 ± 0.019), the activation of channel 28 in MT (0.046 ± 0.020) was higher than in ET (0.041 ± 0.030), and the activation of channel 29 in DT (0.044 ± 0.023) were higher than in ET (0.023 ± 0.013) and MT (0.041 ± 0.010).

#### 3.1.2 Functional connectivity

**FIGURE 4:**
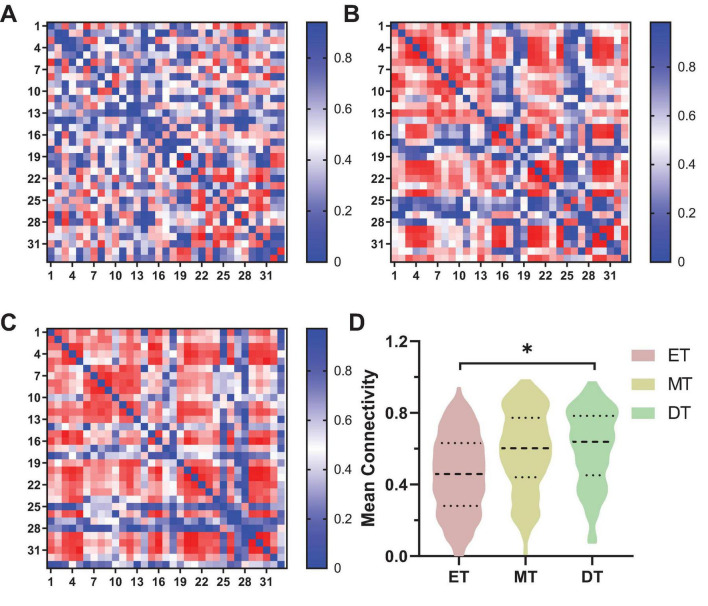
Results of FC of channel-to-channel. *P < 0.05. **(A)** ET, **(B)** MT, **(C)** DT. **(D)** Mean connectivity strength of 33 channels among three groups. ET, easy task; MT, medium task; DT, difficult task.

The connectivity strength between channels for each group is shown in Figure 4. The mean channel-to-channel connectivity strength was 0.46 ± 0.21 for the ET (Figure 4A), 0.58 ± 0.22 for the MT (Figure 4B), and 0.61 ± 0.21 for the DT (Figure 4C). There was a significant difference in FC values among the three groups (*F* = 3.24, *P* = 0.04). The group-averaged FC in the DT was significantly higher than that in the ET (*P* = 0.023; Figure 4D). However, there were no significant differences between the ET and MT (*P* = 0.15) and between the MT and DT (*P* = 0.26).

**FIGURE 5:**
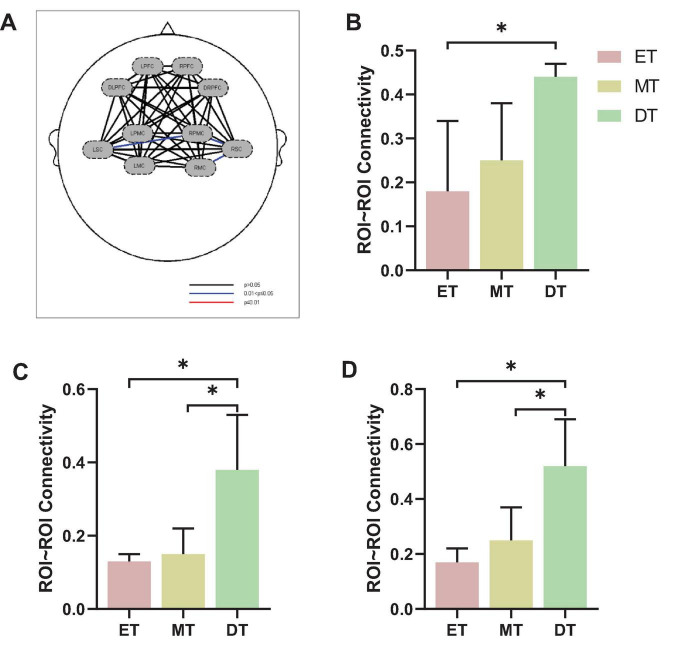
Results of FC of ROI-to-ROI. **(A)** FC of ROI-to-ROI, **(B)** LSC∼RPMC, **(C)** RPMC∼RSC, **(D)** RSC∼RMC. *P < 0.05. ET, easy task; MT, medium task; DT, difficult task; RPMC, right premotor cortex; LSC/RSC, left/right sensorimotor cortex; RMC, right motor cortex.

Figure 5 shows the FC of ROI-to-ROI among the three groups (Figure 5A). Specifically, the FC results showed that the connectivity strength of ET (0.18 ± 0.16) between LSC and RPMC was significantly lower than DT (0.44 ± 0.03, *P* = 0.033; Figure 5B). Additionally, the connectivity strength of DT between RPMC and RSC (0.38 ± 0.15; Figure 5C) and between RSC and RMC (0.52 ± 0.17; Figure 5D) was significantly higher than both ET (*P* = 0.014) and MT (*P* = 0.034).

#### 3.1.3 Laterality index

**FIGURE 6:**
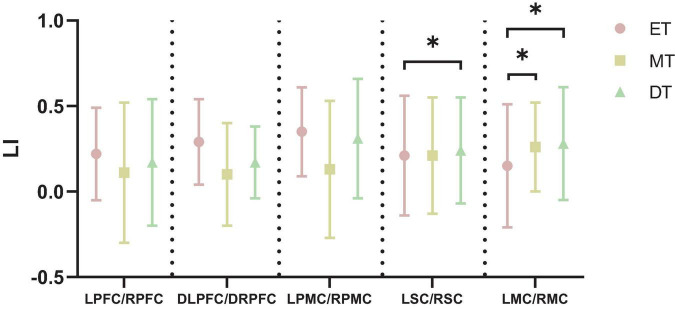
Results of LI. *P < 0.05. ET, easy task; MT, medium task; DT, difficult task; LI, laterality index; LPFC/RPFC, left/right prefrontal cortex; DLPFC/DRPFC, left/right dorsolateral prefrontal cortex; LPMC/RPMC, left/right premotor cortex; LSC/RSC, left/right sensorimotor cortex; LMC/RMC, left/right motor cortex.

Figure 6 shows the cortical activation symmetry. The LI ranged from 0.10 to 0.35, indicating that subjects mainly realized right brain lateralization while performing interactive motor-cognitive dual task. There was a significant difference in the LSC/RSC between ET (0.21 ± 0.35) and DT (0.24 ± 0.31). Additionally, the LI value of LMC/RMC was smaller during ET (0.15 ± 0.36) than during MT (0.26 ± 0.26) and DT (0.28 ± 0.33). No significant differences in LI values were detected between the other groups (*P* > 0.05).

### 3.2 Behavioral data

#### 3.2.1 Walking performance

**FIGURE 7:**
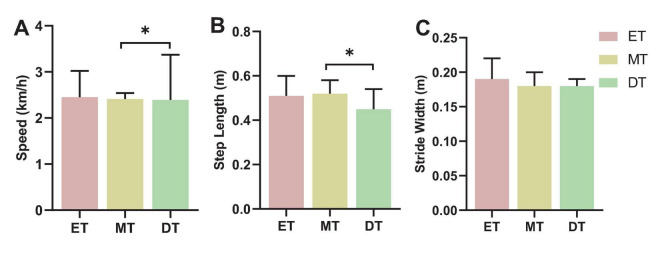
Results of walking performance. *P < 0.05. **(A)** Speed, **(B)** step length, **(C)** stride width. ET, easy task; MT, medium task; DT, difficult task.

The speed (*F* = 8.69, *P* = 0.031; Figure 7A) and stride length (*F* = 2.53, *P* = 0.047; Figure 7B) during DT were significantly smaller than during MT. There was no significant difference in stride width between the three tasks (*F* = 1.73, *P* = 0.062; Figure 7C).

#### 3.2.2 Cognitive performance

**FIGURE 8:**
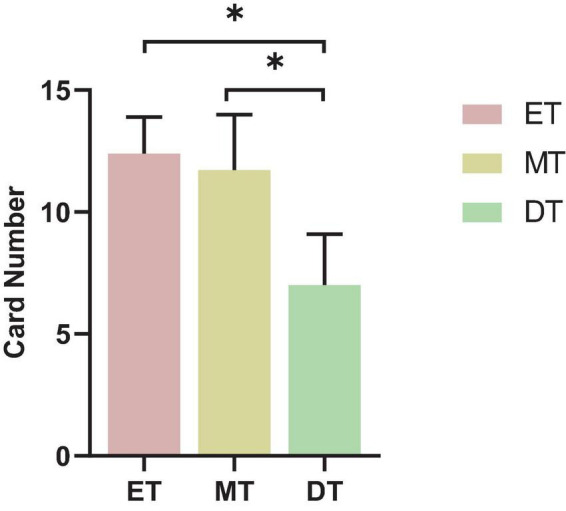
Results of cognitive performance. *P < 0.05. ET, easy task; MT, medium task; DT, difficult task.

The number of words found by the subjects in the DT was lower than in the ET and MT (*F* = 4.55, *P* = 0.042; Figure 8).

## 4 Discussion

This study aimed to explore the impact of varying difficulty levels of interactive cognitive-motor dual task on brain activation, FC, and behavioral performance in healthy adults using fNIRS. By examining the hemodynamic responses across the PFC and motor cortex, significant HbO differences were observed in the DRPFC, LPMC, RPMC, RSC, LMC, and RMC across the ET, MT, and DT conditions. Additionally, FC analysis showed distinct connectivity strengths across these regions as task difficulty increased, particularly between regions such as LSC∼RPMC, RPMC∼RSC, and RSC∼RMC. These results support our initial hypothesis that as task difficulty increased, both brain activation and FC across multiple cortical regions also increased. Our findings reveal notable adaptations in brain activation patterns and FC strength in response to increasing task difficulty, offering valuable insights into the neural mechanisms underlying motor-cognitive integration.

In this study, significant ROI-level activation differences were primarily observed in the DRPFC, LPMC, RPMC, RSC, LMC, and RMC regions. Elevated metabolic activity in the PFC has proven that it is strongly associated with increased planning and attention in motor and cognitive tasks (Hamacher et al., 2015; Wang et al., 2023). The motor cortex plays a critical role in the planning, coordination, and execution of motor functions, which are essential for gait control during dual-task walking (Ou et al., 2024; Rubenstein and Rakic, 2020). Previous studies on dual-task walking have predominantly focused on PFC activation, with findings showing that PFC activation generally increases during dual-task walking compared to single-task walking (Al-Yahya et al., 2019; Chen et al., 2022; Stuart et al., 2018). However, in our study, DLPFC activation during MT was lower than during ET. This discrepancy may be attributed to the specific nature of the motor-cognitive interaction, where the demands on motor coordination might outweigh those on cognitive engagement, supporting the “posture-first” strategy (Bloem et al., 2006). This strategy, in which individuals prioritize the walking task over other concurrent tasks, is typically used by younger individuals but less frequently by older adults (Bloem et al., 2006). In this scenario, motor cortex, rather than the PFC, may take on a more prominent role as the cognitive component is deprioritized. These findings imply that the allocation of cognitive and motor resources could shift depending on task complexity, with some motor-cognitive tasks diverting processing demands from the PFC to motor regions. Furthermore, as task difficulty increased in this study, activation levels in the LPMC/RPMC and LMC/RMC regions also rose, reinforcing this explanation.

Further examination of channel-specific activation revealed significant differences in channels 3, 18, 24, 25, 28, and 29, located in the LPFC, LPMC, RPMC, RSC, and RMC. These findings closely correspond to the activation patterns observed in the ROIs, indicating that task complexity influences not only larger cortical areas but also distinct neural pathways essential for motor-cognitive integration. Channel 3 in the LPFC appears to play a crucial role in higher-order cognitive processing, while the increased activation in channels 18, 24, 25, 28, and 29 likely reflects a more nuanced response within motor cortex regions, facilitating precise motor control under higher task demands. These findings have implications for designing personalized rehabilitation protocols based on channel- and ROI-specific activation patterns. For patients in need of cognitive rehabilitation, exercises can be designed to intensively engage the LPFC, enhancing executive function and cognitive control during physical tasks. Conversely, for patients who require improved motor coordination, activities engaging the LPMC, RPMC, RSC, and RMC can be prioritized to bolster motor control and sensory processing. This targeted approach to rehabilitation may optimize both cognitive and motor function recovery based on individual activation patterns.

Our analysis of FC showed that the average channel-to-channel connectivity strength was lower during the ET compared to the DT, consistent with previous studies indicating that FC strength typically increases with greater task difficulty and cognitive load (Chen et al., 2022; Ding et al., 2024). This suggests a neural recruitment mechanism, where higher task complexity enhances coordination between brain regions to optimize the synchronization of cognitive and motor resources (Chen et al., 2022; Chirles et al., 2017). Interestingly, despite the increase in cognitive task difficulty, we observed stronger FC in motor regions, particularly between LSC∼RPMC, RPMC∼RSC, and RSC∼RMC. This aligns with our activation results, which showed significant engagement of the LPMC, RPMC, RSC, and RMC, suggesting that motor regions play a more prominent role under higher cognitive load. One possible explanation is that greater cognitive demand increases the need for motor planning and coordination, requiring greater motor cortex involvement to maintain gait stability and movement control (Rubenstein and Rakic, 2020). This supports previous research showing that higher task complexity recruits additional motor resources to prevent performance deterioration (Chen et al., 2022; Chirles et al., 2017; Obrig et al., 2000). Additionally, the strengthened FC in motor regions further supports the “posture-first” strategy, where individuals prioritize stable motor execution over cognitive engagement under high cognitive demand. These findings suggest that even when task difficulty is primarily cognitive, the motor system adapts to maintain performance, highlighting the strong interaction between cognitive and motor networks in dual-task conditions.

The LI analysis in this study revealed a dominant role of the right hemisphere during the interactive motor-cognitive dual tasks. Traditionally, the right hemisphere is specialized in functions such as spatial cognition, visual-spatial processing, and motor control (Borrell et al., 2023; Güntürkün et al., 2020). In our dual-task setup, which combined walking with a Stroop task, participants were required to identify both the color and spatial position of words, tasks that heavily rely on spatial processing. This explains the stronger involvement of the right hemisphere, as it aligns with the cognitive demands of the task. Our findings are consistent with recent neuroimaging studies, which have shown right-lateralized activation in the DLPFC during dual-task paradigms involving spatial navigation and walking (Möller et al., 2024; Rahman et al., 2021). This suggests that the right hemisphere plays a crucial role in integrating cognitive and motor functions, particularly when tasks require simultaneous spatial processing and movement control. Additionally, previous studies have suggested that handedness is one of the factors affecting the lateralization pattern (Shirzadi et al., 2020). All participants in this study were right-handed, which is representative of the general population. Research suggests that both right- and left-handed individuals tend to show varying degrees of right-hemisphere dominance for spatial processing (Powell et al., 2012). Some studies further indicate that while right-handed individuals typically rely on the right hemisphere for spatial tasks, left-handed individuals may display less pronounced hemispheric specialization (Shirzadi et al., 2020; Vogel et al., 2003). These findings highlight the complexity of hemispheric dominance and its role in spatial-motor integration.

As task difficulty increased, participants exhibited enhanced FC, yet their behavioral performance declined, with slower gait parameters and reduced word identification. This suggests that although the brain recruits additional neural resources to compensate for increased cognitive load, this adaptation is insufficient to fully counteract dual-task interference. When cognitive demands exceed the brain’s processing capacity, performance declines become inevitable (Chen et al., 2022; Hairu et al., 2021). Several theories explain this phenomenon (Pashler et al., 2001; Wickens, 2002; Wollesen and Voelcker-Rehage, 2014). The central bottleneck theory suggests that a bottleneck in information processing allows only one task to be processed at a time; thus, processing of a second task cannot begin until the first task is complete, often resulting in delayed responses in a dual task setting (Pashler et al., 2001). The four-dimensional multiple resource model posits that interference between tasks increases when they share stages, sensory modalities, processing codes, or visual information channels (Wickens, 2002). Additionally, the attentional resource theory explains declines in motor–cognitive functioning under dual task conditions as stemming from competing demands for attentional resources, leading to interference and reduced performance in one or both tasks (Wollesen and Voelcker-Rehage, 2014). Beyond experimental findings, these deficits have real-world implications, such as an increased risk of falls in older adults or individuals with neurological conditions. While enhanced FC may indicate a neural compensation mechanism, it does not fully offset the behavioral impairments caused by higher cognitive demands. This suggests that under high task loads, the brain’s ability to allocate resources remains constrained. Future research should investigate whether dual task training can improve cognitive-motor integration, potentially reducing interference effects and enhancing both movement stability and cognitive performance in daily life.

This study provides a critical foundation for developing intervention strategies in populations with cognitive impairments. The observed brain activation patterns suggest that personalized dual-task training (e.g., walking combined with cognitive challenges) can be designed to enhance specific brain region functions: LPFC-focused interventions may improve executive function, while premotor and motor cortex training could optimize motor coordination. Additionally, the progressive strengthening of FC with increasing task difficulty supports the implementation of gradual difficulty progression training, which promotes efficient neural resource integration and reduces cognitive-motor interference in daily life. The dominance of the right hemisphere in spatial processing and motor control, highlights its clinical significance for patients with right-hemisphere impairments (e.g., Alzheimer’s disease or right-hemisphere stroke). Future interventions could prioritize spatial-oriented dual tasks that activate the right hemisphere, tailored to individual lateralization patterns, to maximize rehabilitation outcomes.

Despite the valuable insights offered by this study, several limitations should be acknowledged. First, the sample size was relatively small, which may limit the statistical power and generalizability of the results; future studies with larger cohorts are needed to confirm these findings. Second, the study exclusively included healthy young adults, which restricts the applicability of the results to clinical populations. Replicating the study in individuals with neurological impairments would provide greater insight into the potential translational value of the findings for rehabilitation. Finally, although the interactive Stroop-walking paradigm was designed to mimic real-world dual-task situations, it may not fully capture the complexity and unpredictability of everyday environments. Therefore, the ecological validity of the task remains limited and should be further examined in future research.

## 5 Conclusion

We found that increased task difficulty heightened activation in the premotor and motor cortices, with a tendency toward right hemisphere dominance. Higher task difficulty also strengthened FC, particularly in motor-related regions, indicating greater neural coordination. Behaviorally, participants exhibited slower gait parameters and reduced cognitive performance as task complexity increased, highlighting the impact of dual-task interference.

These findings provide valuable insights into the neural mechanisms of motor-cognitive integration. The right hemisphere’s crucial role in spatial processing and motor control highlights the potential benefits of spatially oriented dual-task training for patients with right-hemisphere impairments, such as stroke or Alzheimer’s disease. Furthermore, the observed increase in FC under high cognitive load supports the use of progressive dual-task training to improve cognitive-motor coordination. For individuals with cognitive deficits, interventions targeting the LPFC may enhance executive function, while motor cortex-focused training could improve movement stability. Future studies should investigate these effects in populations with neurological conditions and explore the long-term impact of dual-task training on brain plasticity and functional recovery.

## Data Availability

The raw data supporting the conclusions of this article will be made available by the authors, without undue reservation.
